# Anterior Cruciate Ligament Repair Leads to Improved Patient-Reported Outcomes Compared to Anterior Cruciate Ligament Reconstruction

**DOI:** 10.7759/cureus.60693

**Published:** 2024-05-20

**Authors:** Elan A Karlin, Julia McCann, Brian J Panish, Xue Geng, Linlin Wei, Evan Argintar

**Affiliations:** 1 Orthopedics, MedStar Georgetown University Hospital, Washington, D.C., USA; 2 Department of Biostatistics, Bioinformatics, and Biomathematics, Georgetown University, Washington, D.C., USA; 3 Orthopedics, MedStar Washington Hospital Center, Washington, D.C., USA

**Keywords:** arthroscopy, acl tear, promis, acl reconstruction, acl repair

## Abstract

Introduction

Anterior cruciate ligament (ACL) tears occur frequently in young athletes, and ligament repair and reconstruction are surgical treatments. Although there are suggested benefits for both approaches, there is a lack of direct comparisons between ACL repair and reconstruction.This study aims to compare the mid-term functional outcomes and quality of life measures between patients that have undergone ACL repair versus reconstruction.

Methods

A retrospective review was conducted for demographic and operative report data of patients who underwent an ACL repair or reconstruction between 2012 and 2018. Patients were contacted over the phone and underwent a Patient-Reported Outcomes Measurement Information System (PROMIS) survey evaluating pain interference, mobility, and function. Patients were excluded from the study if there was an incomplete operative note, missing contact information, or failure to answer phone calls.

Results

A total of 74 eligible patients were included, with n = 54 in the ACL reconstruction group (73.0%) and n = 20 in the ACL repair group (27.0%). Reconstruction patients had a PROMIS (median (IQR)) physical function score of 22.50 (16.00-59.00), as compared to repair patients’ physical function score of 60.00 (21.50-60.00). There was a significant difference favoring repair (p = 0.040). In addition, ACL reconstruction patients had a significantly higher rate of additional procedures, with 63.0% of reconstruction patients receiving an additional operation as compared to 30.0% of repair patients (p = 0.017). The surgery type did not show a significant effect on physical function scores, while additional procedures remained significant in the linear regression analysis.

Conclusion

Although ACL repair is associated with improved physical function scores as compared to reconstruction in the univariate analysis, surgery type did not show significance when controlling for other variables. Further studies are necessary to compare patients with similar injuries to account for differences in additional procedures, but the results remain promising in assisting with patient-driven treatment decisions.

## Introduction

Anterior cruciate ligament (ACL) tears are a common injury in young athletes, often requiring operative intervention for treatment. Recent studies have shown that the total incidence of ACL surgeries in the United States stands at nearly 100,000 yearly operations, with a growing prevalence in younger athletes over the past two decades [1-5].

To treat the injury and avoid future complications, ACL repair and reconstruction offer two different options for addressing a torn ACL. The difference between the two approaches lies in reattaching the patient’s own ligament with ACL repair or utilizing tendon autografts or allografts to treat these injuries with ACL reconstruction [6]. Up until the late 20th century, ACL repair was widely considered the preferred protocol for ACL tears, but several clinical trials indicated better patient-reported outcomes and surgical success among patients in the ACL reconstruction groups [7]. ACL reconstruction thus supplanted ACL repair as the primary treatment option and is the “gold standard” [8].

Recent analysis of the primary literature comparing the two treatment options has shown the studies advocating for ACL reconstruction to be lacking consistency in operation timing, technique, and numerous intraoperative and postoperative protocols [7,9]. Combined with the evolving surgical ACL repair technique, the discussion over choosing ACL repair versus reconstruction has become a prominent orthopedic debate, with several studies analyzing the difference in treatment [6,10,11].

Considering the prevalence of ACL tears, along with the lack of consensus among orthopedists in the approach to treating the injury and achieving the best patient-reported outcomes, further study is warranted. The aim of this study is to evaluate patient-reported outcomes as a means of comparison between patients that have undergone either ACL repair or reconstruction with the hypothesis that patients who receive ACL repair will have improved outcomes as compared to ACL reconstruction patients.

This article was previously presented as a meeting abstract at the Virginia Orthopaedic Society on April 28, 2023, and the Maryland Orthopaedic Association on February 11, 2023.

## Materials and methods

Study design

Institutional review board approval from Georgetown Institutional Review Board (Study 3768) was obtained for this study. Patient-reported outcome measures were collected through a Patient-Reported Outcome Measurement Information System (PROMIS) pain interference, mobility, and function survey via phone interviews. Informed consent was obtained prior to conducting the questionnaire.

The PROMIS survey functions through computer-adaptive testing and has been proven to be a reliable health-related quality-of-life measure while providing precise outcome measures [12,13]. It has been used in the literature to monitor outcomes of ACL tears and treatment and has been shown to be without the ceiling effects found in other commonly employed patient-reported outcome instruments [14,15].

**Figure 1:**
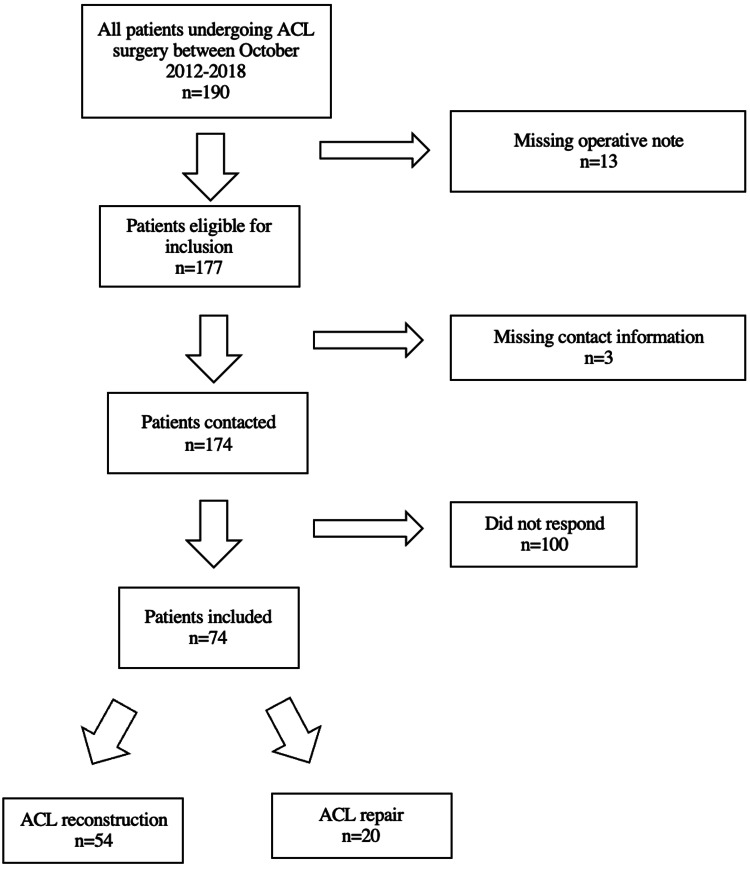
Flowchart demonstrating the studied ACL repair and reconstruction populations ACL: anterior cruciate ligament

Patient demographic data and operative notes were retrospectively collected through Medstar Georgetown University Hospital’s electronic health records to obtain information pertaining to the date of surgery, graft type (for ACL reconstruction), BMI, and additional, concomitant surgical procedures. Patients were included in the study if they underwent an ACL repair or reconstruction from October 2012 to October 2018 by the principal investigator. In total, there were 190 patients who underwent ACL surgery (Figure 1).

Exclusion criteria consisted of the following: missing operative note, missing contact information, or failure to complete the phone questionnaire. The final analysis included 74 patients with 54 ACL reconstruction and 20 ACL repair patients. The decision for ACL repair versus reconstruction was made intraoperatively by the attending surgeon and senior author based on tear location and tissue quality.

Surgical Technique

Both ACL reconstruction and repair were performed arthroscopically with standard anteromedial and anterolateral arthroscopy portals. Diagnostic arthroscopy was performed, the ACL was evaluated, and the decision was made for either ACL reconstruction or repair by the senior author. All proximal ACL tears that had good tissue quality were repaired (Sherman Type I) [16]. Tissue quality was assessed by the surgeon in the following manner: after the ACL was tagged, the sutures were pulled vigorously. Repair continued if after this stress, there was no ligament degradation or significant change to the tagging suture. All other ACL injuries were treated with reconstruction.

For ACL reconstruction, autograft (bone-patellar tendon-bone, hamstring, and quadricep) or allograft tendons (peroneal, hamstrings) were utilized. Graft selection was done after shared decision-making. The advantages of each type were discussed with the patient. The grafts were prepared using the same standard technique, and femoral and tibial tunnels were drilled anatomically using a FlipCutter (Arthrex, Inc.; Naples, FL). Proximal fixation was a TightRope (Arthrex, Inc.; Naples, FL) and distal fixation was an ABS button (Arthrex, Inc.; Naples, FL). All ACLs had internal brace augmentation, with distal fixation using a SwiveLock (Arthrex, Inc.; Naples, FL), as supported by evidence-based medicine [9]. Tensioning was performed at 30 degrees.

**Figure 2:**
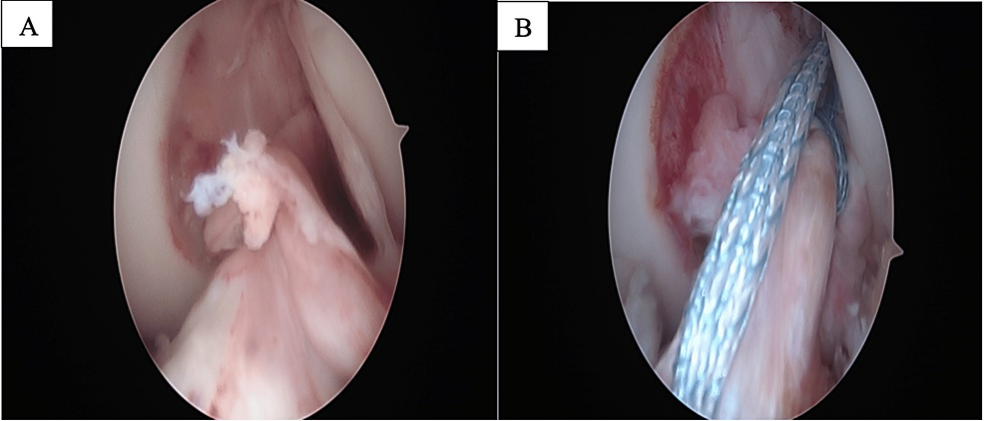
Images taken from the repair of the ACL tear. View from the lateral inferior patellar arthroscopy portal. The patient is supine and the knee flexed 90°. A: demonstrating the ACL tear. B: ACL repair with an internal brace.

ACL repair was performed after the native ACL was tagged with FiberWire (Arthrex, Inc.; Naples, FL). This suture, along with a fiber tape looped through a TightRope (Arthrex, Naples, Fl), was passed through a femoral tunnel drilled at the ACL origin. Fiber tape internal bracing was then passed through the tibia at the insertion of the ACL. Internal brace fixation was accomplished using a SwiveLock (Arthrex, Inc.; Naples, FL). Proximal fixation was performed by tying the suture attached to the ACL ligament to the suture in the button of the tightrope. Tensioning was performed at 30 degrees [17] (Figure 2). 

The postoperative rehabilitation protocol was identical in both cohorts, with full range of motion and weight bearing initiated immediately and formal physical therapy begun two weeks after surgery.

Data Collection and PROMIS

The patients were contacted by phone between two years and eight years postoperatively to complete the PROMIS survey, evaluating pain interference, mobility, and function. Raw PROMIS scores were calculated based on patient responses, with higher scores indicating more of the outcome of interest (i.e., more mobility or more pain interference). 

Statistical analysis

Statistical analysis was performed by biostatisticians of the Department of Biostatistics at our home institution. Data normality was checked by the Shapiro-Wilk test. Data were overall summarized by frequency and percentage for categorical variables and median and interquartile range (IQR) for continuous variables. 

The univariate associations between outcomes and surgery type (repair and reconstruction), gender (male and female), laterality (right and left), autograft (bone-patellar tendon-bone, hamstring, quadricep) or allograft (peroneal, hamstrings) type, additional procedure (yes and no), and continuous variables (age at surgery, BMI) were checked. Based on the normality of variables, a two-sided Wilcoxon rank-sum test was used to compare continuous variables between 520 groups. The Kruskal-Wallis test was used to compare continuous variables between three or more groups.

A chi-square test was used to check the association between two categorical variables, and Fisher’s exact test was used instead when the expected cell had fewer than five subjects. Moreover, the two-sided Spearman’s correlation was employed to check the association between two continuous variables. A linear regression model was built for each outcome to evaluate the effect of surgery type (repair vs. reconstruction) on PROMIS scores adjusting for gender (male vs. female), laterality (right vs. left), age at surgery, body mass index (BMI), and additional procedure (yes vs. no). All tests are at a significant level of 0.05. All analyses were performed using the statistical software RStudio (version 1.4.1106, RStudio Team, Integrated Development for R. RStudio, PBC, Boston, MA).

## Results

Study population

A total of 74 eligible patients were included in the study with 20 out of 29 eligible patients in the repair group and 54 out of 145 eligible patients in the reconstruction group (Figure 1). The average follow-up time for all patients was 4.9 years postoperatively. For the repair group, the average follow-up time was 3.7 years, while the reconstruction group had an average follow up of 5.3 years. Patients had a PROMIS (median (IQR)) pain interference score of 12.00 (7.00-12.00), mobility score of 60.00 (24.00-60.00), and physical function score of 23.00 (16.00-60.00). The median age at surgery was 31.43 (26.91-35.24), while the median body mass index was 25.16 (23.47-27.64). Median and IQR values are reported as none of the continuous variables were normally distributed. Thirty-two patients were female (43.2%), and 42 patients were male (56.8%). The left knee was operated on in 32 cases, while 42 of the total operations (43.2%) were performed on the right knee (56.8%). Forty patients received an additional, concomitant procedure (54.1%), while 34 did not receive an additional procedure (45.9%). 

Reconstruction and repair populations

Of the patients in the study, 54 were included in the ACL reconstruction group (73.0%) and 20 in the ACL repair group (27.0%) (Table 1). Reconstruction patients had a PROMIS pain interference score of 12.00 (6.25,12.00), mobility score of 60.00 (22.00-60.00), and physical function score of 22.50 (16.00-59.00) (Table 2). The median BMI (IQR) and age for reconstruction patients were 25.21 (23.72-27.45) and 30.11 (26.02-33.53), respectively. Of the 54 reconstruction patients, 23 were female (42.6%) and 31 were male (57.4%). Thirty-four patients received an additional, concomitant procedure (63.0%). 

**Table 1:** Baseline patient characteristics N (%) or median (IQR) Demographic data for patients included in the study. Variables of interest include age, sex, BMI, additional procedure, and laterality. * = statistically significant, p <0.05.

Variable	Reconstruction	Repair	p-value
	N = 54	N = 20	
Age, median	30.11 (26.02–33.53)	33.14 (31.05-38.24)	0.041*
Sex			0.99
Male	23 (42.6)	9 (45.0)	
Female	31 (57.4)	11 (55.0)	
BMI	25.21 (23.72, 27.45)	25.15 (22.98, 30.17)	0.948
Additional procedure			0.02*
Yes	34 (63.0)	6 (70.0)	
No	20 (37.0)	14 (30.0)	
Laterality			0.937
Left	24 (44.4)	8 (40.0)	
Right	30 (55.6)	12 (60.0)	

**Table 2:** PROMIS subscore median (IQR) raw score values and corresponding p-values when compared between the ACL reconstruction and repair groups. Using 𝛼=0.05, there was a statistically significant difference in mid-term physical function subsets favoring ACL repair over reconstruction. * = statistically significant, p <0.05.

PROMIS Score (median (IQR))	Reconstruction	Repair	p-value
Pain interference	12.00 [6.25, 12.00]	12.00 [9.00, 12.00]	0.984
Mobility	60.00 [22.00, 60.00]	60.00 [59.75, 60.00]	0.092
Physical function	22.50 [16.00, 59.00]	60.00 [21.50, 60.00]	0.040*

Repair patients had a PROMIS pain interference score of 12.00 (9.00,12.00), mobility score of 60.00 (59.75-60.00), and physical function score of 60.00 (21.50-60.00). The median (IQR) BMI and age for repair patients were 25.15 (22.98-30.17) and 33.14 (31.05-38.24), respectively. Of the 20 repair patients, nine were female (45.0%) and 11 were male (55.0%). Six patients received an additional procedure (30.0%).

Outcomes

There was a statistically significant difference in the physical function score between the repair and reconstruction groups favoring repair (p = 0.040). There was no statistically significant difference between the two groups for pain interference (p = 0.984) and mobility (p = 0.092) subscores. There was no significant difference in pain interference, physical function, or mobility scores when comparing allograft and autograft subtypes within the reconstruction group. ACL reconstruction patients had a significantly higher rate of additional, concomitant procedures at 63% as compared to 30% of repair patients (p = 0.02) (Table 3). There was also a statistically significant difference in age at the time of the procedure, with a younger reconstruction group (p = 0.041). In addition, univariate association analysis showed a statistically significant association between BMI and mobility raw score, with a correlation coefficient of -2.80 (p = 0.018). When controlling for covariates including surgery type, gender, laterality, additional procedure, BMI, and age at surgery, there was no longer a statistically significant difference between ACL repair and reconstruction in physical function scores. However, when controlling for other variables, patients who had additional procedures had a statistically significant decrease in the physical function raw score by 14.91 (95% CI: -24.86 to -4.96) compared to patients who did not have any additional procedures (p = 0.005).

**Table 3:** Concomitant procedures for patients included in the study. * = statistically significant, p <0.05.

Concomitant procedure	ACL reconstruction	ACL repair	p-value
Meniscectomy	15 (27.8)	2 (10)	0.049*
Meniscal repair	15 (27.8)	2 (10)	0.049*
Anterior lateral ligament reconstruction	2 (3.7)	1 (5.0)	0.021*
Microfracture	3 (5.6)	0 (0.0)	0.046*
Loose body removal	2 (3.7)	0 (0.0)	0.036*
Cartilage biopsy	1 (1.9)	0 (0.0)	0.030*
Removal of hardware	2 (3.7)	0 (0.0)	0.036*
Chondroplasty	1 (1.9)	1 (5.0)	0.009*
Patellar tendon repair	1 (1.9)	0 (0.0)	0.030*

## Discussion

This study demonstrates comparable patient-reported outcome measures between ACL repair and reconstruction groups. Although the groups were not matched for concomitant procedures or age, ACL repair patients were shown to have a statistically significant improvement in their physical function as compared to ACL reconstruction patients after mid-term follow-up.

This study compared the mid-term functional outcomes and quality of life measures between ACL repair and reconstruction patients using PROMIS scores. PROMIS scores have been shown to provide an efficient, accurate, and reliable measure of both physical and mental health with advantages over existing summary scores [12-15]. Split into three raw scores (mobility, pain interference, and physical function), the PROMIS system can isolate the reasons for patient dissatisfaction following their operation. In addition, the use of PROMIS for ACL repair and reconstruction has been validated through comparison to previously used patient-reported outcome measures [18].

For the past several decades, ACL reconstruction has been considered the primary form of ACL tear treatment. The prevalence of ACL tears, combined with 70% of injured patients facing secondary knee osteoarthritis, requires reexamination of the preferred method of treatment [8,19].

Studies have shown several benefits of the ACL repair approach, including a less invasive surgery, chondroprotective benefits, maintaining better motion, and providing faster recovery over its counterpart [9,20]. Additional proposed benefits include greater proprioception and hamstring muscle strength [10,21,22]. Furthermore, other studies have shown the long-term association between postoperative muscle weakness and osteoarthritis among ACL reconstruction patients with time [9,23]. However, others have defended reconstruction as the standard for ACL treatment, citing lower complication rates and less residual anteroposterior knee joint laxity [24,25]. It is evident there is a lack of clarity on the superior approach in the field of orthopedics. Despite the extensive assessment between ACL repair and reconstruction, most of the literature lacks direct comparison in the primary measure of surgical success, patient-reported outcomes [5,9,20-22]. The studies that have considered the patient’s satisfaction with their knee’s function and pain lack extended follow-up [11,26,27].

In addition, the development of improved ACL repair techniques necessitates further analysis. Both Köster et al. and Häberli et al. studied the dynamic intraligamentary stabilization (DIS) ACL repair technique and compared its outcomes to ACL reconstruction populations. Both groups of researchers found similar success in revision and failure rates, along with functional results [21,28]. Furthermore, other studies have indicated that the surgical success of ACL reconstruction has been overestimated. Enrolling 958 patients over the span of 12 years, Rousseau et al. found a startling total complication rate of 39% and revision rate of 28%, indicating the need for caution and prolonged follow-up when treating ACL reconstruction patients [29]. Considering these studies that cite similar complication rates, along with others that defend the improved muscle strength and function among ACL repair patients, it may be ill-advised to view ACL reconstruction as the standard for ACL tears [9,10,21-23].

Despite the interest in comparing ACL repair and reconstruction patients, the current literature lacks longer follow-up time of patient-reported outcome measures [6,9,11,20-22]. Vermeijden et al. were one of the first groups to consider a mid-term approach to patients’ perception of their knee post-operation. Analyzing 83 patients after two to five years post-surgical intervention, the researchers found a statistically significant difference in forgotten joint scores between ACL repair and reconstruction patients. The ACL repair patients had better outcomes with regard to noticing any abnormality with their joints [30]. 

The results of this study suggest that ACL repair leads to improved patient outcomes compared to ACL reconstruction when considering physical function scores at mid-term follow-up. In addition, patients with a higher BMI were associated with lower mobility scores. This is in congruence with several studies indicating that elevated BMI can restrict the mobility of joints [31-33]. Further studies are necessary to compare patients with similar injuries and age-matched groups, but the results remain promising in assisting with patient-driven treatment decisions.

Limitations

There are several limitations to our study. First, the study is retrospective in nature and carries with it the associated limitations. This includes relying on patients to recall outcomes and the inability to determine causation, only association. The decision between ACL repair versus reconstruction was made intraoperatively by the attending surgeon. Since judgment was based on surgical experience, this may have introduced a source of bias. 

As mentioned, a greater percentage of ACL reconstruction patients had an additional procedure as compared to the ACL repair group. Specifically, there were more CPT codes associated with reconstruction surgeries. Future studies will aim to compare ACL repair and reconstruction with similar additional procedure rates. Moreover, this study lacked preoperative PROMIS scores. Future studies should collect preoperative scores to analyze improvements in the outcome between the repair and reconstruction groups. The patient population for this study was small, necessitating future studies with larger sample sizes. We suspect that research fatigue and our patient population at an urban level one trauma center limited patient interest in participation, leading to poor patient follow-up. The majority of the patients who chose not to participate in this study had in fact participated in several other investigations and had demonstrated outcomes consistent with standard ACL reconstruction surgery [9,34-40].

Despite these limitations, this study is still one of the first, to our knowledge, to consider and show mid-term differences in PROMIS scores following ACL reconstruction and repair performed by a single surgeon.

## Conclusions

Our study indicates that ACL repair is associated with mid-term improved PROMIS physical function scores as compared to ACL reconstruction. Further research is necessary to compare patients with similar concomitant injuries, but the results remain promising in assisting with patient-driven treatment decisions. To our knowledge, this study is one of the first to show mid-term differences in patient-reported outcomes following ACL repair versus reconstruction.
